# Antihyperglycemic Effects and Mode of Actions of *Musa paradisiaca* Leaf and Fruit Peel Hydroethanolic Extracts in Nicotinamide/Streptozotocin-Induced Diabetic Rats

**DOI:** 10.1155/2020/9276343

**Published:** 2020-01-26

**Authors:** Sarah M. Abdel Aziz, Osama M. Ahmed, Sanaa M. Abd EL-Twab, Hessah Mohammed Al-Muzafar, Kamal Adel Amin, Mohamed Abdel-Gabbar

**Affiliations:** ^1^Biochemistry Division, Chemistry Department, Faculty of Science, Beni-Suef University, P.O. Box 62521, Beni-Suef, Egypt; ^2^Physiology Division, Zoology Department, Faculty of Science, Beni-Suef University, P.O. Box 62521, Beni-Suef, Egypt; ^3^Chemistry Department, College of Science, Imam Abdulrahman Bin Faisal University, P.O. Box. 1982, Dammam 31441, Saudi Arabia; ^4^Basic & Applied Scientific Research Center, Imam Abdulrahman Bin Faisal University, P.O. Box 1982, Dammam, 31441, Saudi Arabia

## Abstract

The present study aimed to evaluate the antihyperglycemic effects of *Musa paradisiaca* (*M. paradisiaca*) leaf and fruit peel hydroethanolic extracts and to suggest their probable mode of actions in nicotinamide (NA)/streptozotocin (STZ)-induced diabetic rats. The leaf and fruit peel hydroethanolic extracts were analyzed by GC-MS that indicated the presence of phytol, octadecatrienoic acid, hexadecanoic acid, and octadecadienoic acid as major components in the leaf extract and vitamin E, octadecenamide, *β*-sitosterol, and stigmasterol as major phytochemicals in the fruit peel extract. Diabetes mellitus was induced by a single intraperitoneal injection of STZ (60 mg/kg body weight) dissolved in citrate buffer (pH 4.5), 15 minutes after intraperitoneal injection of NA (120 mg/kg body weight). The NA/STZ-induced diabetic rats were, respectively, treated with *M*. *paradisiaca* leaf and fruit peel hydroethanolic extracts at a dose of 100 mg/kg body weight/day by oral administration for 28 days. The treatment of NA/STZ-induced diabetic rats with leaf and fruit peel extracts significantly improved the impaired oral glucose tolerance and significantly increased the lowered serum insulin and C-peptide levels. The HOMA-IR (as the index of insulin resistance) and QUICKI (as a marker for insulin sensitivity), as well as HOMA-*β* cell function were significantly alleviated as a result of treatment of diabetic rats with leaf and fruit peel extracts. In association, the elevated serum-free fatty acids, TNF-*α*, and IL-6 levels were significantly decreased. In addition, the suppressed adipose tissue PPAR*γ*, GLUT4, adiponectin, and insulin receptor *β*-subunit mRNA expressions were upregulated while the elevated adipose tissue resistin expression was downregulated in diabetic rats as a result of treatment with the leaf and peel extract. Based on these results, it can be concluded that *M. paradisiaca* leaf and fruit peel hydroethanolic extracts have antihyperglycemic effects which may be mediated via their insulinotropic and insulin-sensitizing effects.

## 1. Introduction

Diabetes mellitus (DM) is an endocrinological disorder [1–3] which is a group of metabolic or heterogeneous affliction resulting from an irregularity in insulin secretion and insulin action or both consistent with derangement in carbohydrate, protein, and lipid metabolism [4]. Thus, the impaired insulin secretion and action in turn lead to persistent abnormally high blood glucose level and glucose intolerance [5]. Characteristically, the disease is responsible for increased risk of cardiovascular diseases including atherosclerosis, renal failure, blindness, or diabetic cataract [6]. According to the International Diabetes Federation (IDF), 425 million people worldwide or 8.8% of adults (20–79 years) are estimated to have diabetes and by 2045, the number would be 629 million of people 20–79 years with a prevalence of 9.9% [7]. With respect to Egypt, the IDF listed Egypt among the world top 10 countries in the number of patients with diabetes, and the number of people with diabetes in Egypt is around 8.2 million individuals and it is expected that this number will jump up to 16.7 million by 2045 [7].

Insulin resistance is the main defect associated with metabolic syndrome and obesity [8]. The adipose tissue plays a central role in insulin sensitivity and energy expenditure [9], and the dysfunction in adipocytes is associated with insulin resistance and type 2 DM (T2DM) [10]. Adipose tissue modulates metabolism by releasing nonesterified fatty acids (NEFAs) and glycerol, hormones including leptin and adiponectin, and proinflammatory cytokines [11, 12]. In obesity, the production of many of these products is increased [10, 13]. Reduced level of adiponectin and increased levels of interleukin-6 (IL-6), tumor necrosis factor-*α* (TNF-*α*), free fatty acids, and resistin can reduce insulin-mediated glucose uptake due to insulin resistance [14].

Since none of the antidiabetic drugs could give a long-term glycemic control without producing any unfavorable side effects, medicinal plants which are effective in improving plasma glucose level with minimal side effects are widely used in underdeveloped and developing countries as an alternative therapy [15]. Of these medicinal plants widely used in the field of herbal medicine, *Musa paradisiaca* (*M*. *paradisiaca*) has been reported to have many advantageous effects in the control of several diseased conditions, including atherosclerosis, DM, hyperlipidemia, hypertension, and thyroid dysfunctions [16, 17] and also produces protective effects on organs of the body, such as the kidneys, in certain clinical conditions [18]. Vijayakumar et al. [19] reported antioxidant efficacies of the isolated flavonoids from *M. paradisiaca* in rats. The *M. paradisiaca* green fruit has been reported to have antihyperglycemic effects due to stimulation of insulin production and glucose utilization [20] in diabetic mice. Its elevated potassium (K) and sodium (Na) content has been correlated with the glycemic state [21]. Fibers from the *M. paradisiaca* fruit enhanced glycogenesis in the liver and decreased fasting blood glucose concentration [22].

Therefore, the current study was designed to scrutinize the antihyperglycemic effect and to suggest the probable mechanism of actions of *M. paradisiaca* peel and leaf hydroethanolic extracts in NA/STZ-induced diabetic rats.

## 2. Materials and Methods

### 2.1. Plant Materials

Fruits of the banana tree (*M. paradisiaca*) were purchased from a local market, and the leaves were collected from the banana agriculture garden, Beni-Suef, Egypt, in the month of May. The plant was authenticated by Dr. Mohamed A. Fadl, Associate Professor of Taxonomy, Botany Department, Faculty of Science, Beni-Suef University, Egypt. Plant samples were deposited in the Herbarium of Botany Department, Faculty of Science, Beni-Suef University, Egypt. By using the NCBI taxonomy database, the taxonomy ID was found to be 89151 (NCBI: txid89151) (https://www.ncbi.nlm.nih.gov/Taxonomy/).

### 2.2. Preparation of Plant Extract

After the botanical authentication, the plant leaves and fruits were washed under running tap water to remove adhering dusts. The fruits were manually peeled, and good-quality peels were selected. The plant materials were air dried under shade and powdered by an electric grinder. The resulting powder materials of leaves and fruit peels were separately subjected to extraction with 70% ethanol. About 500 gram (g) of powdered material of *M. paradisiaca* leaves and fruit peels were soaked in adequate volume of ethanol (70%) for 72 hours with continuous shaking and stirring at room temperature. The solutions were then filtered through muslin cloth. Each obtained filtrate was evaporated to semisolid mass by using a rotary evaporator to obtain the hydroethanolic extract. The viscous green and brown hydroethanolic extract of leaves and peels, respectively, were obtained and stored at −20°C till further use [23]. The yield of the hydroethanolic extracts of leaves and fruit peels was, respectively, 2.8% and 3% of dry weight.

### 2.3. Gas Chromatography-Mass Spectrometry (GC-MS) Analysis

Both hydroethanolic extracts of *M. paradisiaca* leaf and fruit peel extracts were subjected to GC-MS analysis in the Central Laboratory of the Faculty of Postgraduate Studies for Advanced Sciences, Beni-Suef University, Egypt, for the identification of phytochemical components. The GC-MS uses the principle of separation technique. The sample was analyzed in an Agilent 7890A Gas Chromatography (GC) system equipped with a HP-5 MS column (30 m × 250 *µ*m, 0.25 *µ*m film thicknesses) and Agilent 5975C inert Mass Spectrometer Detector with triple-axis detection. Sample at volume 1 *µ*l was injected with the splitless mode. The injection port was maintained at a temperature of 250°C. The temperature program in the oven starts at 120°C, then increases at a rate of 5°C/minute to 220°C, followed by 8°C/minute to 280°C for 5 minutes. Helium gas was used as a carrier gas at a flow rate of 1.0 ml/minute, and the total run time was 32.5 minutes.

### 2.4. Identification of Phytochemical Components

Interpretation of mass spectrum GC-MS results was conducted using the database of National Institute Standard and Technology (NIST) having more than 62,000 patterns and Fiehn MS Libraries. The obtained spectrum of the unknown component was compared with the spectrum of the known components stored in the NIST library (C:\Database\NIST11.L; C:\Database\demo.l). The name, molecular weight, and structure of the components of the test materials were ascertained.

### 2.5. Chemicals

Streptozotocin (STZ), [2-deoxy-2-(3-methyl-3nitrosoureido)-D-glycopyranoside], as a diabetogenic agent and nicotinamide (NA) [pyridine-3-carboxamide] were purchased from Sigma Chemical Co., St Louis, MO, USA. All other chemicals were of analytical grade and were obtained from standard commercial supplies.

### 2.6. Experimental Animals and Ethics Statement

Forty male albino rats weighing about 120–150 g were used as experimental animals in the present study. The rats were obtained from the animal house of the Research Institute of National Research Center (NRC), Doki, Giza, Egypt. They were maintained under follow-up for 15 days before the beginning of the experiment to exclude any intercurrent infection. The chosen animals were maintained in polypropylene cages at temperature (25 ± 5°C), humidity (55 ± 5%), and 12 hours light/dark cycle as well as under good ventilation and was given tap water and standard balanced diet *ad libitum*. The animal procedures were conducted according to the principles and guidelines of the Canadian Council on Animal Care [24] and following the guidelines and instructions of the Experimental Animal Ethics Committee, Faculty of Science, Beni-Suef University, Egypt (ethical approval number: BSU/FS/2016/13).

### 2.7. Induction of Animal Model of DM

Experimental T2DM was induced in overnight-fasted rats by a single intraperitoneal (i.p.) injection of STZ (60 mg/kg b.wt) prepared in citrate buffer (pH 4.5), 15 minutes after the i.p. injection of NA (120 mg/kg b.wt) dissolved in 0.9% saline solution [25]. Seven days after STZ injection, the blood glucose concentrations were determined in the rats to detect the hyperglycemic state. Overnight-fasted (10–12 hours) animals were given glucose (3 g/kg b.wt.) by oral gavage. After 2 hours of oral glucose administration, blood samples were taken from the lateral tail vein and centrifuged. Then, serum glucose concentration was detected. Rats that have a 2-hour serum glucose level ranging from 200 to 300 mg/dl were considered mild diabetic and were included in the experiment.

### 2.8. Animal Grouping

The rats included in the experiment were randomly allocated into 4 groups as follows:

- Group 1: this group was considered as the normal control. The rats in this group were given an equivalent volume of the vehicle (1% CMC), by oral gavage daily for 4 weeks.
- Group 2: this group was considered as the diabetic control. The rats in this group were given an equivalent volume of 1% CMC, by oral gavage daily for 4 weeks.
- Group 3: the rats in this group were diabetic rats treated with the *M. paradisiaca* leaf hydroethanolic extract (100 mg/kg b.wt/day dissolved in 1% CMC) by oral gavage for 4 weeks [26].
- Group 4: the rats in this group were treated with the *M. paradisiaca* peel hydroethanolic extract (100 mg/kg b.wt/day dissolved in 1% CMC) by oral gavage for 4 weeks [26].

### 2.9. Sample Collection

By the end of the fourth week, the animals were starved for 12 hours. Blood samples were collected from the jugular vein under inhalation anesthesia. Blood samples were left at room temperature to coagulate and then centrifuged by cooling centrifuge at 3000 rounds per minute (rpm) for 15 minutes. The clear nonhemolysed sera were kept at -20°C for pending analysis of some biochemical parameters. Immediately after decapitation by cervical dislocation and dissection, visceral adipose tissues were dissected out and weighed. Then, they are stored immediately in RNA later and frozen at −80°C for their pending use in the detection of mRNA expression of various genes using quantitative real-time PCR (qRT-PCR).

### 2.10. Biochemical Examinations

On the day before sacrifice and dissection, the oral glucose tolerance test (OGTT) was performed using blood samples withdrawn from the lateral tail vein of rats that were deprived of food and water overnight (10–12 hours). Following the administration of glucose solution (3 g/kg b.wt), successive blood samples were then taken at 0, 30, 60, 90, and 120 minutes. Blood was left to coagulate and then centrifuged at 3000 rpm for 15 minutes. The supernatant serum of blood from each rat was aspirated into Eppendorf tubes for determination of various biochemical parameters. Serum glucose level was estimated according to the method of Trinder [27] by using a reagent kit purchased from Spinreact (Spain). Serum levels of insulin and C-peptide were assayed by Sandwich ELISA using kits purchased from Linco Research (USA), according to the manufacturer instructions. TNF-*α* and IL-6 were determined using specific ELISA kits (R&D systems) following the manufacturer's instructions. Serum-free fatty acids (FFAs) level was estimated according to the method of Duncombe [28]. Because abnormalities in insulin action are poorly detected by a single determination of glucose or insulin levels [29], insulin resistance and insulin sensitivity were, respectively, evaluated by homeostasis model assessment-insulin resistance (HOMA-IR) [30] and quantitative insulin-sensitivity check index (QUICKI) [31] as follows:

  HOMA − IR = fasting insulin level (*μ*U/ml) × fasting blood glucose (mmol/L)/22.5,
  QUICKI = 1/log fasting insulin level (*μ*U/ml) + log fasting blood glucose (mg/dl).

HOMA-*β* cell function was calculated according to Kuang et al. [32] as follows:

  HOMA-*β* cell function = (20 × fasting insulin [*μ*IU/ml]) ÷ ([fasting glucose (mg/dl) ÷ 18)] − 3.5).

RNA isolation and quantitative real time-polymerase chain reaction (qRT-PCR): Gene expression analysis of peroxisome proliferator-activated receptor gamma (PPAR*γ*), adiponectin, glucose transporter type 4 (GLUT4), resistin, and insulin receptor *β*-subunit in the adipose tissue samples was performed. Total RNA was isolated from visceral adipose tissue samples by a method using the TRIzol® reagent (Invitrogen, Carlsbad, CA, USA) according to the manufacturer′s instructions and treated with RNAse-free DNAse (Invitrogen). Purified RNA was quantified spectrophotometrically at 260 nm using a NanoDrop ND-1000 spectrophotometer (Thermo Fisher scientific, Waltham, USA), and RNA purity was checked by means of the absorbance ratio at 260/280 nm. In addition, RNA integrity was assessed by electrophoresis on 2% agarose gel. Reverse transcription was performed with 1 *μ*g RNA using the Reverse Transcription System (Promegs, Leiden, Netherlands). The resulting cDNA was amplified using the SYBR Green master mix (Applied Biosystem) in a total volume of 20 *μ*l using the primer sets listed in Table 1. The qPCR reaction was performed in an optical 96-well plate with an ABI PRISM 7500 fast sequence detection system (Applied Biosystems, Carlsbad, California) and with the following universal cycling conditions: initial denaturation at 95°C for 10 minutes, 40 cycles of denaturation at 95°C for 15 seconds, annealing at 60°C for 60 seconds, and extension at 72°C for 30 seconds; a final step at 60°C, increased about 0.5°C every 10 seconds up to 95°C. Data were analyzed with the ABI Prism sequence detection system software and quantified using the v1.7 Sequence Detection Software from PE Biosystems (Foster City, CA). Each experiment included a distilled water control. The amplification data were analyzed following the 2-ΔΔCt method [33], and the values were normalized to *β*-actin.

### 2.11. Statistical Analysis

Statistical analysis was performed using Statistical Package for Software Package, SPSS version 20 [34]. Results were represented as mean ± standard error (SE), and all statistical comparisons were made by means of one-way ANOVA test followed by Tukey's test post hoc analysis. *P* values > 0.05 were considered nonsignificantly different while those of *P* < 0.05 were significantly different.

## 3. Results

**Figure 1:**
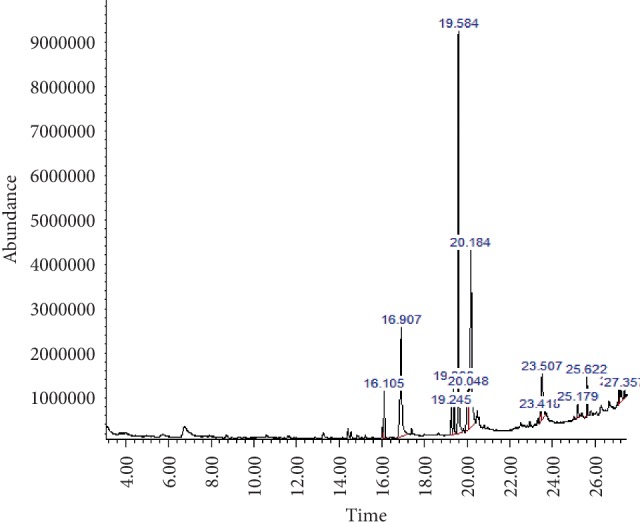
GC-MS analysis of the hydroethanolic *M. paradisiaca* leaf extract.

**Figure 2:**
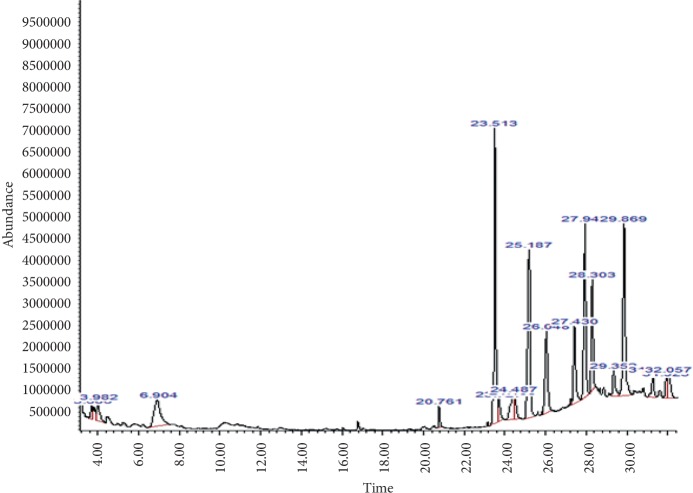
GC-MS analysis of the hydroethanolic *M. paradisiaca* peel extract.

GC-MS analysis the *M. paradisiaca* hydroethanolic leaf and fruit peel extract showed the presence of several phytocomponents. The identified phytocomponents with their retention time (RT), molecular formula (M/F), molecular weight (MW), and relative abundance, which was expressed as peak area% (%PA), and their activity are summarized in Tables 2 and 3 and depicted in Figures 1 and 2.

**Figure 3:**
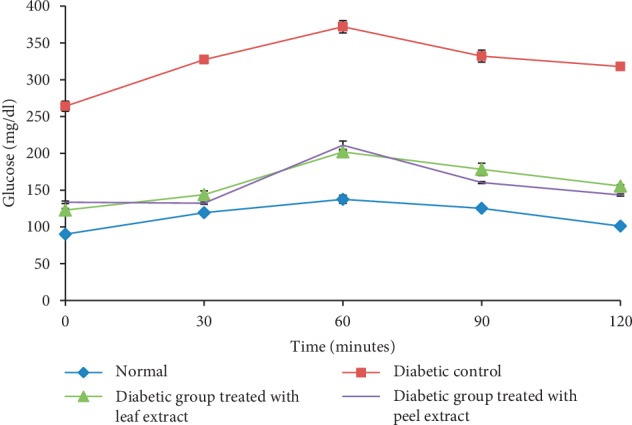
OGTT of normal, diabetic control, and diabetic rats treated with *M. paradisiaca* leaf and fruit peel hydroethanolic extracts.

NA/STZ-induced diabetic rats showed a significant increase (*P* < 0.05) in serum glucose levels at all tested time intervals of OGTT after oral glucose loading (3 glucose/kg b.wt) as compared to normal rats (Figure 3). Oral administration of either *M. paradisiaca* leaf or fruit peel hydroethanolic extracts to diabetic rats significantly (*P* < 0.05) reduced the elevated blood glucose levels at all points of the OGTT.

**Figure 4:**
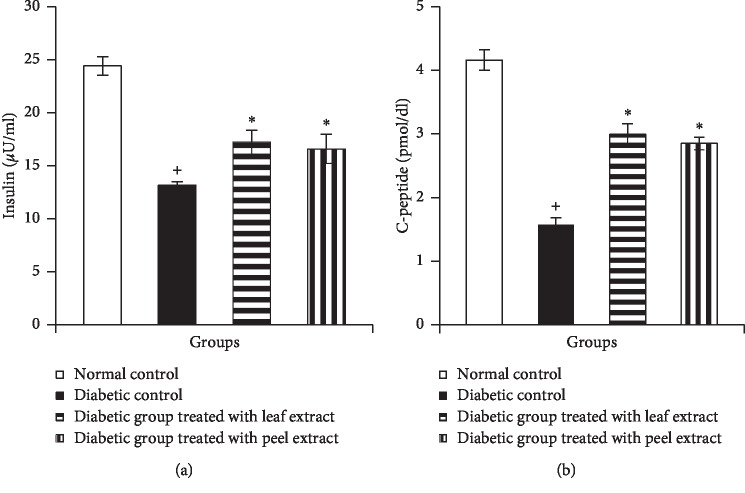
Serum insulin (a) and C-peptide (b) levels in normal, diabetic control, and diabetic rats treated with *M. paradisiaca* leaf and fruit peel extracts. ^+^Significant as compared with the normal control. ^*∗*^Significant as compared with the diabetic control.

Diabetic rats revealed a significant (*P* < 0.05) decline in serum insulin and C-peptide levels when compared with normal control rats (Figures 4(a) and 4(b)). Oral treatment of diabetic rats with the *M. paradisiaca* leaf extract as well as fruit peel hydroethanolic extract significantly (*P* < 0.05) improved serum insulin and C-peptide levels.

**Figure 5:**
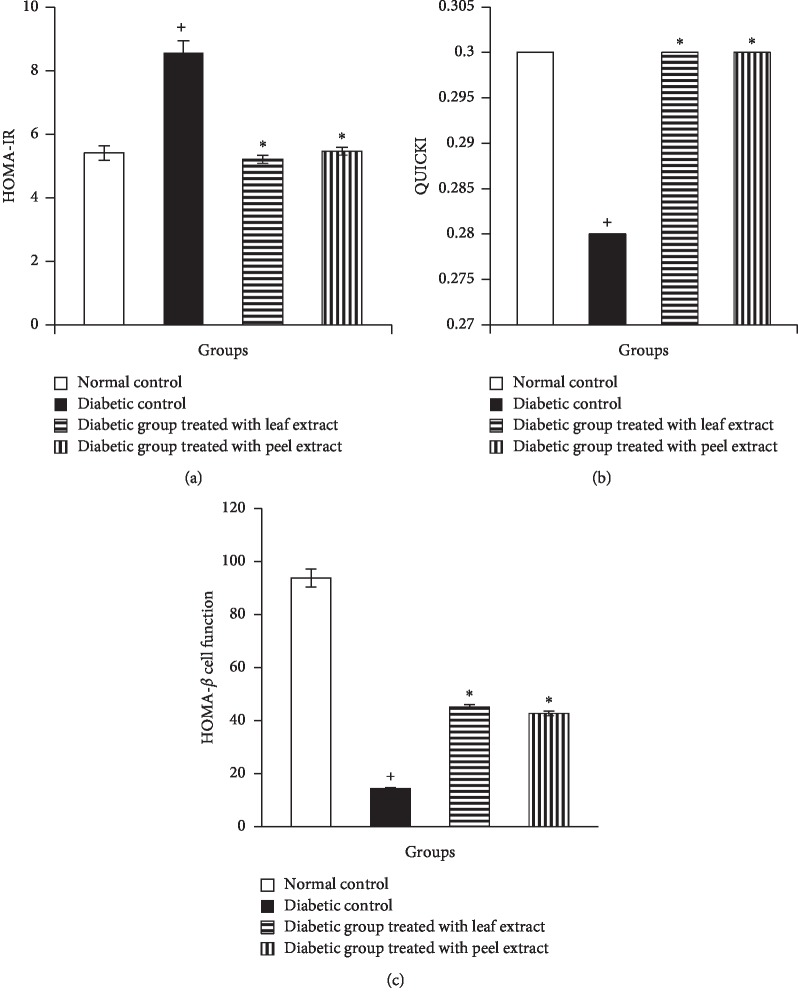
HOMA-IR index (a), QUICKI (b), and HOMA-*β* cell function (c) in normal, diabetic control, and diabetic rats treated with *M. paradisiaca* leaf and fruit peel extracts. ^+^Significant as compared with the normal control. ^*∗*^Significant as compared with the diabetic control.

To assess the effect of *M. paradisiaca* leaf and fruit peel hydroethanolic extracts on insulin resistance and sensitivity, HOMA-IR and QUICKI were determined. Diabetic rats exhibited a significant (*P* < 0.05) increase in HOMA-IR and decrease in QUICKI. The treatment with *M. paradisiaca* leaf and fruit peel extracts produced a significant (*P* < 0.05) amelioration in insulin sensitivity as evident by their effect on HOMA-IR and QUICKI (Figures 5(a) and 5(b)). To assess *β*-cell function and secretory response, HOMA-*β* cell function was calculated and it exhibited a significant (*P* < 0.05) decrease in diabetic rats. The treatment with *M. paradisiaca* leaf and fruit peel hydroethanolic extracts produced a significant increase in HOMA-*β* cell function, but it failed to return to normal level (Figure 5(c)).

**Figure 6:**
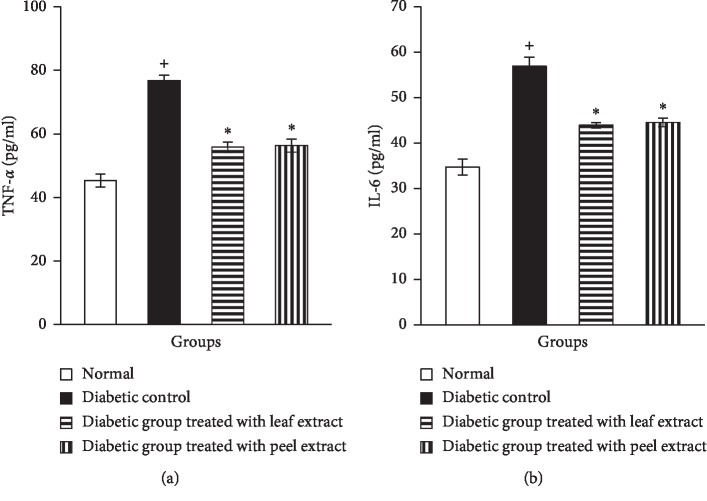
Serum TNF-*α* (a) and IL-6 (b) levels in normal, diabetic control, and diabetic rats treated with *M. paradisiaca* leaf and fruit peel extracts. ^+^Significant as compared with the normal control. ^*∗*^Significant as compared with the diabetic control.

The levels of the proinflammatory cytokines, TNF-*α* and IL-6, in the serum of NA/STZ-induced diabetic rats showed a significant (*P* < 0.05) elevation when compared with the corresponding control group. Oral supplementation with *M. paradisiaca* leaf and fruit peel hydroethanolic extracts produced a significant (*P* < 0.05) decrease in serum TNF-*α* and IL-6 levels in diabetic rats (Figures 6(a) and 6(b)).

**Figure 7:**
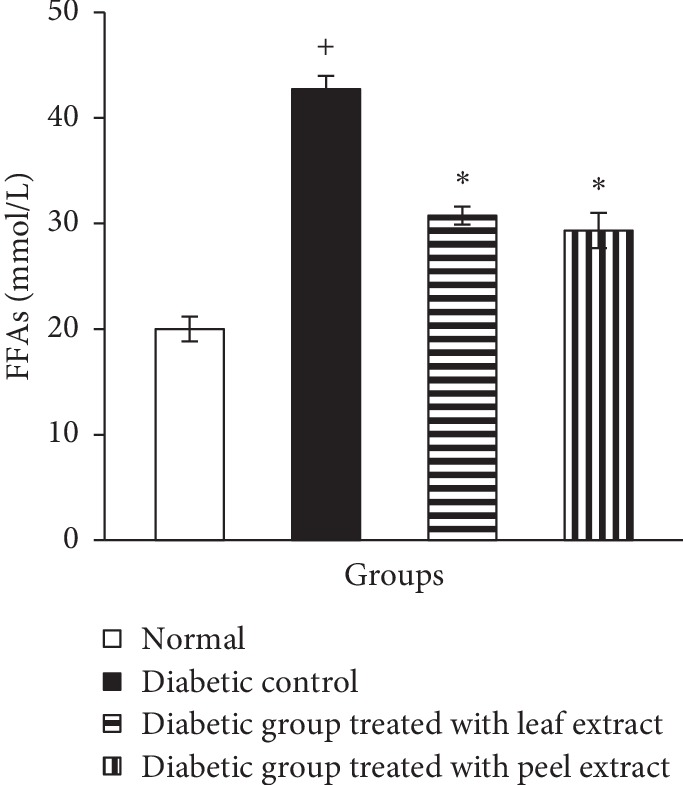
Serum FFAs level in normal, diabetic control, and diabetic rats treated with *M. paradisiaca* leaf and fruit peel extracts. ^+^Significant as compared with the normal control. ^*∗*^Significant as compared with the diabetic control.

Data on the effect of *M. paradisiaca* peel and leaf extract on FFAs of diabetic rats are presented in Figure 7. The diabetic rats displayed a significant increase (*P* < 0.05) in serum FFAs as compared with the nondiabetic group. The administration of both *M. paradisiaca* leaf and fruit peel hydroethanolic extracts led to a significant reduction (*P* < 0.05) of the elevated levels of serum FFAs.

**Figure 8:**
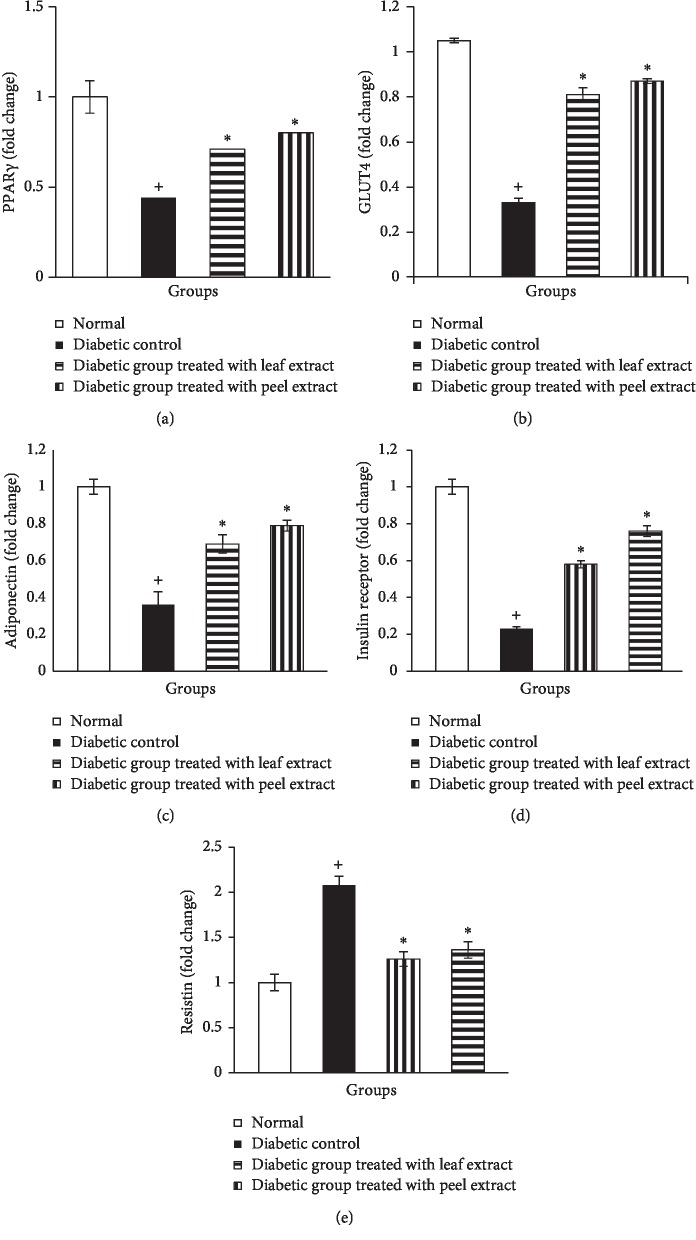
Adipose tissue mRNA expression of PPAR*γ* (a), GLUT4 (b), adiponectin (c), insulin receptor-*β* subunit (d), and resistin (e) in normal, diabetic control, and diabetic rats treated with *M. paradisiaca* leaf and fruit peel extracts.

Adipose tissue mRNA expression of PPAR*γ*, GLUT4, adiponectin, and insulin receptor *β*-subunit revealed a significant (*P* < 0.05) decrease in NA/STZ-induced diabetic rats when compared with the normal control group (Figures 8(a)–8(d)). On the contrary, supplementation of the diabetic rats with the *M. paradisiaca* leaf and fruit peel hydroethanolic extracts produced a significant (*P* < 0.05) increase; the fruit peel extract was more potent (Figures 8(a)–8(d)). In contrast, adipose resistin mRNA expression was significantly (*P* < 0.05) elevated in NA/STZ-induced diabetic rats in comparison with the normal and was significantly downregulated (*P* < 0.05) by the treatment of diabetic rats with *M. paradisiaca* leaf and fruit peel hydroethanolic extracts. The treatment with the peel extract seemed to be more potent in decreasing the elevated resistin mRNA expression (Figure 8(e)).

## 4. Discussion

T2DM is the most common form of DM characterized by elevated blood glucose levels, insulin resistance, and relative insulin deficiency [36]. Indeed, appropriate experimental animal models have provided important information on metabolic, genetic, and environmental risks of diabetes and helped to scrutinize the molecular mechanisms underlying the development, progression, and therapeutic control of this disease [37]. In the present study, the NA/STZ diabetic rat model, with abnormal glucose tolerance and insulin activity, was used to investigate the antidiabetic activity of *M. paradisiaca* peel and leaf hydroethanolic extracts. The STZ/NA rat model of T2DM is based on the preventive effects of NA against *β*-cytotoxic effects of STZ [38]. However, if NA is injected prior to STZ, the severity of DM will be attenuated to a certain extent, leading to a T2DM-like condition with impaired insulin sensitivity [39, 40].

Phytochemical constituents are responsible for medicinal activity of plant species [41]. Hence, in the present study, phytochemical components in the hydroethanolic extract of *M*. *paradisiaca* leaf and fruit peel extracts contain phytol, octadecatrienoic acid, hexadecanoic acid, and octadecadienoic acid as major components in the leaf extract and vitamin E, octadecenamide, *β*-sitosterol, and stigmasterol as major phytochemicals in the fruit peel extract. Most of these phytochemicals have been already reported as exerting hypoglycemic, hypolipidemic, and antioxidant effects [42–45].

In our study, the NA/STZ-induced diabetic rats revealed a significant elevation in serum glucose level in association with higher HOMA-IR values as compared with the normal control. However, serum insulin and C-peptide levels as well as QUICKI and HOMA-*β* cell function were significantly diminished in diabetic rats as compared to the control ones. Therefore, this rat model not only exhibits hyperglycemia and insulin deficiency, but also shows insulin resistance that would closely reflect the natural history and metabolic characteristics of human T2DM and is further sensitive to pharmacological testing.

Oral treatment of diabetic rats, in the current study, with hydroethanolic extracts of *M. paradisiaca* leaves and fruit peels significantly ameliorated serum glucose level. The observed decrease in the elevated glucose levels is in accordance with studies of Hongmei et al. [46] who reported that the banana peel and its ingredient lupenone showed promising antihyperglycemic activity. The antihyperglycemic effects of *M. paradisiaca* leaf and fruit peel extracts may be attributed to their ability to enhance both insulin secretion and insulin action. This explanation was supported by our results which revealed a significant increase in serum insulin and C-peptide levels as well as QUICKI (as marker of insulin sensitivity) and HOMA-*β* cell function and a significant decrease in HOMA-IR (as the index of insulin resistance) due to treatments of diabetic rats with *M. paradisiaca* leaf and fruit peel extracts.

In the present study, the treatment of NA/STZ-induced diabetic rats with of *M. paradisiaca* leaf and fruit peel hydroethanolic extracts significantly decreased the elevated serum TNF-*α* and IL-6 levels confirming their antiinflammatory efficacy. It is worth mentioning that TNF-*α* is a potent proinflammatory cytokine primarily secreted from myeloid cells via activation of MAPK and NF-*κ*B signaling pathways, resulting in the release of other inflammatory cytokines, such as IL-1*β* and IL-6 [47]. TNF-*α* elicits antagonic activity towards insulin because of its ability to augment the insulin receptor substrate-1 (IRS-1) and insulin receptor phosphorylation on serine or threonine residues [48]. Altered insulin receptor substrate (IRS) and insulin receptor phosphorylation on serine or threonine reduces the phosphorylation of tyrosine residues through protein kinase C and the nuclear factor kappa B (NF-*κ*B), a regulatory protein kinase (I*κκβ*), and prevented the activation of phosphatidylinositol 3-kinase (PI3K) [49, 50] and protein kinase B (Akt/PKB) [51, 52]. The reduction in tyrosine phosphorylation causes insulin resistance [53]. TNF-*α* also downregulates the mRNA levels of adiponectin [54], which contributes to the maintenance of peripheral glucose and lipid homeostasis [55]. TNF-*α* has been found to markedly increase lipolysis and FFA release, at least in part through a reduced perilipin expression [56] and decreased Gi protein expression [57] further augmenting the impaired cellular insulin signaling and glucose uptake [56]. Thus, the decrease in the FFAs levels, the increase in adipose tissue mRNA expression, and the enhancement in insulin sensitivity, in the present study, as a result of treatment of NA/STZ-induced diabetic rats with *M. paradisiaca* leaf and fruit peel hydroethanolic extracts may be secondary to the improvement in the elevated TNF-*α* level.

IL-6 plays a direct role in insulin resistance in skeletal muscles as well as in the liver due to the defects in IRS phosphorylation resulting in decreased gluconeogenesis and increased glycogenolysis [58]. It contributes to dyslipidemia via the expression of microsomal triglyceride transfer proteins that help in the assembly of apolipoprotein B in the liver [59]. IL-6 has been shown to activate SOCS-1 and -3 proteins in the liver, thus accompanying insulin resistance [58, 60]. Based on these evidences, it can be suggested that the amelioration of blood IL-6 level may have a role in the improvement of insulin sensitivity as a result of treatment of NA/STZ-induced diabetic rats with *M. paradisiaca* leaf and fruit peel hydroethanolic extracts.

The antiinflammatory effects of *M. paradisiaca* leaf and fruit peel hydroethanolic extracts may be attributed to the presence of phytochemicals such as phytol, vitamin E, and phytosterols such as *β*-sitosterol and stigmasterol. These compounds have been reported to exert antiinflammatory effects by several authors [61–63].

In the current study, the serum elevated FFAs level in NA/STZ-induced diabetic rats was significantly decreased due to treatments with *M. paradisiaca* leaf and fruit peel hydroethanolic extracts. FFAs regulate gene expression especially those involved in lipid and carbohydrate metabolism [64]. The mechanisms by which the elevated levels of FFAs decrease insulin sensitivity include inhibition of insulin-stimulated glucose transport [65], lipotoxicity hypothesis [66] that results in impairment of insulin secretory function through toxic effects on pancreatic *β*-cells, and finally increased lipolysis of the visceral adipose tissue and subsequent flux of FFAs to the nonadipose tissue leading to excessive endogenous glucose production and progression of insulin resistance and T2DM [67]. Thus, the decrease in serum FFAs in the diabetic rats treated with *M. paradisiaca* leaf and fruit peel hydroethanolic extracts is participating in their insulin-sensitizing effects and improved *β*-cells secretory response.

In association with the decrease in serum fatty acid level, the treatment of NA/STZ-induced diabetic rats with *M. paradisiaca* leaf and fruit peel hydroethanolic extracts resulted in an increase in the expression of adipose tissue PPAR*γ*, GLUT4, adiponectin, and insulin receptor *β*-subunit.

PPARs modulate expression of the genes involved in metabolism of lipids [68, 69]. Its activation stimulates lipid oxidation and lipogenesis, induces differentiation of adipocytes, and increases insulin sensitivity in mature adipocytes [70]. Therefore, synthetic PPAR*γ* ligands such as thiazolidinediones (TZDs) are applied clinically to control diabetes [71]. PPAR*γ* increases the expression and translocation of GLUT4 in the adipose tissue, increases the catabolism of glucose in the liver along with the reduction in the hepatic glucose output [72], and decreases the insulin resistance in the muscle [73]. The action of PPAR*γ* on insulin sensitivity results from its ability to channel fatty acids (FAs) into the adipose tissue, thus decreasing plasma FAs concentration and alleviating lipotoxicity in the skeletal muscle, liver, and pancreas [74, 75]. In addition, PPAR*γ* can affect insulin sensitivity by regulating adipocyte hormones, cytokines, and proteins that are involved in insulin resistance. Indeed, PPAR*γ* downregulates the expression of genes encoding resistin and tumor necrosis factor (TNF-*α*), whereas it induces adiponectin expression, which increases fatty acid oxidation by activation of the AMP-activated protein kinase pathway [75, 76].

GLUT4 is a member of the glucose transporter family that exists in many tissues specifically in the skeletal muscle and adipose tissues [77], which play a critical role in the insulin-stimulated glucose transport in these tissues, with glucose uptake occurring when insulin stimulates the translocation of GLUT4 from the intracellular pool to the plasma membrane [78]. The increased GLUT4 expression in diabetic rats treated with *M. paradisiaca* leaf as well as fruit peel hydroethanolic extracts may be due to the enhanced insulin secretory response of *β*-cells and the improved peripheral insulin-sensitizing effects of both agents.

Adiponectin, one of the most important adipokine, increases *β*-oxidation of FFAs in muscles and glucose transport mediated by phosphorylation of AMPK [79] and inhibition of acetyl-CoA carboxylase [80], inhibits hepatic gluconeogenesis secondary to decreasing the expression of phosphoenolpyruvate carboxylase and glucose-6-phosphatase [81], and increases fatty acid combustion and energy consumption, partly through PPAR*α* activation, leading to decreased triglyceride content in skeletal muscles and the liver [82]. Therefore, adiponectin may be a local regulator for glucose utilization in the adipocytes and adipose tissue via its regulation of PPAR*γ*, glucose, and lipid transcriptional factor expression [81, 83]. It is well established that there is an inverse relationship between insulin resistance and plasma adiponectin levels, suggesting that adiponectin is an important regulator of insulin sensitivity and glucose homeostasis [84, 85], possibly through stimulation of AMP-activated protein kinase (AMPK) [82]. Based on the previous elucidation and on our results, the antidiabetic effect of the hydroethanolic extract of *M. paradisiaca* leaves and fruit peels in NA/STZ-induced diabetic rats might be explained, at least in part, through its ability to produce a pronounced increase in mRNA expression of adiponectin.

Similar to the effects on adipose tissue PPAR*γ*, GLUT4, and adiponectin, the treatment of diabetic rats with hydroethanolic extracts of *M. paradisiaca* leaf and fruit peel extracts induced upregulation for insulin receptor *β*-subunit gene expression. The mechanism may involve increased expression of key protein insulin receptor, insulin receptor substrate-1 (IRS-1), and phosphatidylinositol 3-kinase (PI3K)) that are involved in the insulin-signaling processes. Insulin receptor is a disulfide-linked protein composed of two *α* and two *β* subunits present in the plasma membrane of target cells [86]. The extracellular *α* subunits contain the ligand-binding domain, and the intrinsic *β* subunits contain the tyrosine kinase-signaling domains. Not only does the insulin receptor regulate lipid metabolism through suppression of lipolysis and induction of lipogenesis in adipocytes, but also stimulates glucose uptake [87]. Insulin resistance is partly mediated by reducing levels of insulin receptor expression [88]. This leads to impaired tyrosine phosphorylation of the insulin receptor and subsequent tyrosine phosphorylation of IRS-1 and reduced P85/PI3K activity in response to insulin.

Consistent with the impaired glucose tolerance, HOMA-IR, and QUICKI in NA/STZ-induced diabetic rats, the present results showed a significant elevation in adipose tissue resistin mRNA expression as compared to the control group. These results are in accordance with Rajala et al. [89] who demonstrated that circulating resistin levels were significantly elevated and positively concordant with rising levels of insulin, glucose, and lipids in Lep ob/ob mice. In addition, Kim et al. [90] have reported that resistin is expressed exclusively in adipocytes and is linked with the traits that are related to obesity and insulin resistance. These findings may be attributed to resistin-induced impairment of glucose homeostasis and insulin action that modulates one or more steps in the insulin signaling pathway and likely participates in the pathogenesis of insulin resistance [91]. Different impacts of resistin on insulin sensitivity have been suggested. This include decreasing the phosphorylation of AMPK [91, 92], increasing the suppressor of cytokine signaling 3 (SOCS-3) expression [93], decreasing activation of PPAR*γ* [94], regulating NF-*κ*B expression [95], and suppressing GLUT4 gene expression [96]. Resistin induced the elevation of endogenous glucose production, as indicated by the upregulation of insulin-independent expression of genes encoding the hepatic gluconeogenic enzymes glucose-6-phoshatase and phosphoenolpyruvate carboxykinase in liver cells [97] and downregulation of glycogen synthase activity [98]. Also, it promotes lipid accumulation in human macrophages by upregulating CD36 cell surface expression, which is one of the scavenger receptors in macrophages involved in the uptake of modified LDL [99]. Hydroethanolic extracts of *M. paradisiaca* leaf and fruit peel extracts' supplementation to diabetic rats significantly downregulated adipose tissue resistin expression. Therefore, the potent antidiabetic effect of *M. paradisiaca* in the present study could perhaps be attributed, at least in part, to its resistin-modulating effect.

## 5. Conclusion

**Figure 9:**
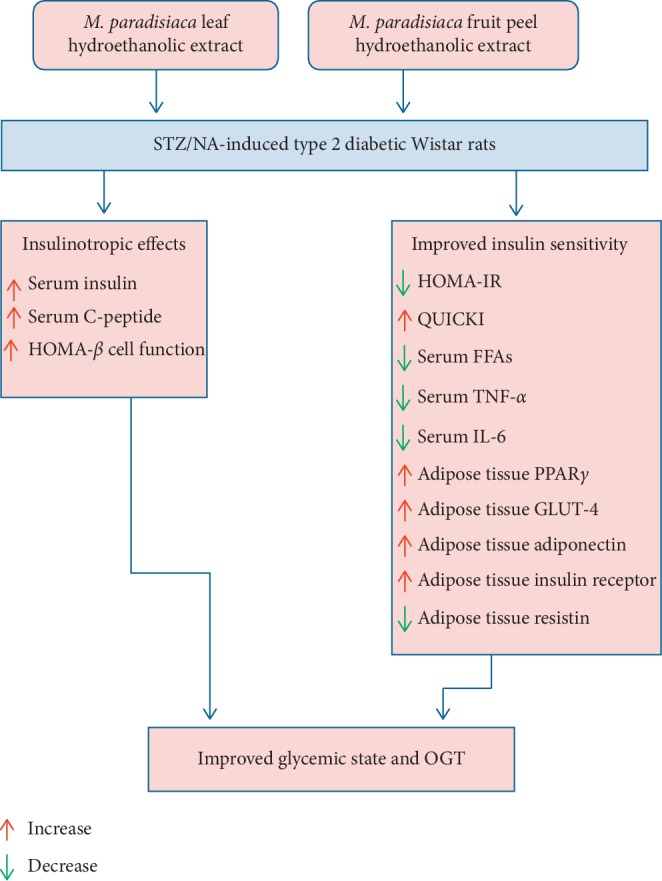
Schematic diagram showing the mode of actions of *M. paradisiaca* leaf and peel hydroethanolic extracts in NA/STZ-induced type 2 diabetic rats.

Oral administration of *M. paradisiaca* leaf and fruit peel hydroethanolic extracts potentially improves the glycemic state in NA/STZ-induced diabetic rats via improving the insulin secretory response of *β*-cells and peripheral tissue insulin sensitivity. The insulin-sensitizing activities of both extracts may be mediated through downregulation of proinflammatory cytokines (TNF-*α* and IL-6), FFAs, and adipose tissue expression of resistin and upregulation of adipose tissue PPAR*γ*, GLUT4, adiponectin, and insulin receptor expression (Figure 9).

## Figures and Tables

**Table 1:** Primer sequence used for qRT-PCR.

Gene	Forward	Reverse
PPAR_γ_	5′GGACGCTGAAGAAGAGACCTG3′	5′CCGGGTCCTGTCTGAGTATG3′
GLUT4	5′GCTGTGCCATCTTGATGACGG3′	5′TGAAGAAGCCAAGCAGGAGGAC3′
Adiponectin	5′AATCCTGCCCAGTCATGAAG3′	5′CATCTCCTGGGTCACCCTTA3′
Resistin	5′GCTCGTGGGACATTCGTGA3′	5′CGGGCTGCTGTCCAGTCTA3′
Insulin receptor (*β*-subunit)	5′TGTGGCAAGAGCCAAAGGAA3′	5′TTCCCATGCCTTGGTCTCCT3′
*β*-Actin	5′TACAACCTTCTTGCAGCTCCT 3′	5′CCTTCTGACCCATACCCACC3′

**Table 2:** Bioactive compounds present in the *M. paradisiaca* hydroethanolic leaf extract.

Leaf extract							
No.	RT	Name of the compound	Molecular formula	MW	Peak area %	Compound nature	Activity
1	16.104	Hexadecanoic acid, methyl ester	C_17_H_34_O_2_	270	3.46	Palmitic acid ester	Antioxidant, antiandrogenic, pesticide, antihypercholesterolemic, nematicide, lubricant, flavor, and hemolytic
2	16.90	n-Hexadecanoic acid	C_16_H_32_O_2_	256	16.35	Palmitic acid	Antioxidant, antiinflammatory, antihypercholesterolemic, hemolytic, nematicide, pesticide, antiandrogenic, flavor, and 5-alpha reductase inhibitory
3	19.24	9,12-Octadecadienoic acid (Z,Z)-, methyl ester	C_19_H_34_O_2_	294	1.99	Linolenic acid	Antiinflammatory, hypocholesterolemic cancer preventive, hepatoprotective, nematicide insectifuge, antihistaminic antieczemic, antiacne, 5-alpha reductase inhibitor antiandrogenic, antiarthritic, and anticoronary
4	19.36	9,12,15-Octadecatrienoic acid, methyl ester, (Z,Z,Z)-	C_19_H_32_O_2_	292	3.81	Linoleic acid ester	Antihypercholesterolemic, 5-alpha reductase inhibitor, nematicide, antiarthritic, hepatoprotective, and antiandrogenic
5	19.58	Phytol	C20H40O	296	30.59	Diterpene	Antimicrobial, antiinflammatory, anticancer, and diuretic
6	20.04	9,12-Octadecadienoic acid (Z,Z)-	C_18_H_32_O_2_	294	6.20	Linolenic acid	Antiinflammatory, nematicide, insectifuge, antihistaminic, antiacne, antihypercholesterolemic, cancer chemopreventive, hepatoprotective, antiarthritic, antieczemic, 5-alpha reductase inhibitor, antiandrogenic, and anticoronary
7	20.18	9,12,15-Octadecatrienoic acid, methyl ester, (Z,Z,Z)-	C_19_H_32_O_2_	292	23.98	Linolenic acid ester	Hypocholesterolemic, nematicide, antiarthritic, hepatoprotective, antiandrogenic, and 5-alpha reductase
8	23.41	9-Octadecenamide, (Z)-	C_18_H_35_NO	281	0.74	Oleamide	Antioxidative, antiepileptic, neuroprotective, and hypolipdmic
9	23.50	9-Octadecenamide, (Z)-	C_18_H_35_NO	281	5.74	Oleamide	Antiepileptic, neuroprotective, hypolipidmic, and antioxidative
10	25.17	No match	—	—	1.35	—	—
11	25.62	Bis(2-ethylhexyl) phthalate	C_24_H_38_O_4_	390	2.24	Phthalic acid ester	No activity reported
12	27.10	No match	—	—	1.46	—	—
13	27.188	No match	—	—	1.59	—	—
14	27.36	No match	—	—	0.49	—	—

Modified from Dr. Duke's Phytochemical and Ethnobotanical Databases [35].

**Table 3:** Bioactive compounds present in the *M. paradisiaca* hydroethanolic fruit peel extract.

Peel extract							
No.	RT	Name of the compound	Molecular formula	MW	Peak area %	Compound nature	Activity
1	3.688	No matches	—	—	0.91	—	—
2	3.980	No matches	—	—	1.72	—	—
3	6.903	No matches	—	—	5.78	—	—
4	20.76	Hexadecanamide	C_16_H_33_NO	255	0.92	Palmitamide	Energy source
5	23.51	9-Octadecenamide, (Z)-	C_18_H_35_NO	281	15.97	Oleamide	Hypolipdmic, antioxidative, antiepileptic, and neuroprotective
6	23.72	9-Octadecenamide, (Z)	C_18_H_35_NO	281	1.26	Oleamide	Hypolipdmic, antioxidative, antiepileptic, and neuroprotective
7	24.40	Campesterol	C_28_H_48_O	400	2.51	Sterols	Antiangiogenic, antiinflammatory, antioxidant, anticancer, and cholesterol-lowering activity
8	24.48	Ergost-5-en-3-ol, (3.beta.)-	C_28_H_48_O	400	2.01	Sterols	Antimicrobial, antiinflammatory, anticancer, antihypercholesterolemic, and cardioprotective
9	25.18	Vitamin E	C_29_H_50_O_2_	430	15.16	Vitamin E	Antiageing, analgesic, antidiabetic, antiinflammatory, antioxidant, antidermatitic, antileukemic, antitumor, anticancer, hepatoprotective, hypocholesterolemic, antiulcerogenic, vasodilator, antispasmodic, antibronchitic, and anticoronary
10	26.04	Stigmasterol	C_29_H_48_O	412	7.43	Sterols	Antidiabetic, anticancer, antiinflammatory, hypocholestrolemic, and antioxidant
11	7.43	Campesterol	C_28_H_48_O	400	2.51	Sterols	Antiangiogenic, antiinflammatory, antioxidant, anticancer, and cholesterol-lowering.
12	27.93	*β*-Sitosterol	C_29_H_50_O	414	11.79	Sterols	Anticholesteremic, antidiabetic, antioxidant, anticancer, antiinflammatory, antiarthritic, antiasthma, and diuretic
13	28.30	Stigmasterol	C_29_H_48_O	412	7.16	Sterols	Antidiabetic, anticancer, antiinflammatory, hypocholestrolemic, and antioxidant
14	29.351	No matches	—	—	2.2	—	—
15	29.87	*β*-Sitosterol	C_29_H_50_O	414	13.49	Sterols	Anticholesterolemic, antidiabetic, antioxidant, anticancer, antiinflammatory, antiarthritic, antiasthma, and diuretic
16	31.268	No matches	—	—	1.83	—	—
17	31.926	No matches	—	—	1.12	—	—
18	32.057	—	—	—	2.51	—	—

Modified from Dr. Duke's Phytochemical and Ethnobotanical Databases [35].

## Data Availability

All data used in this article are publicly available and accessible online.
